# Intramyocardial Transplantation and Tracking of Human Mesenchymal Stem Cells in a Novel Intra-Uterine Pre-Immune Fetal Sheep Myocardial Infarction Model: A Proof of Concept Study

**DOI:** 10.1371/journal.pone.0057759

**Published:** 2013-03-22

**Authors:** Maximilian Y. Emmert, Benedikt Weber, Petra Wolint, Thomas Frauenfelder, Steffen M. Zeisberger, Luc Behr, Sebastien Sammut, Jacques Scherman, Chad E. Brokopp, Ruth Schwartländer, Viola Vogel, Peter Vogt, Jürg Grünenfelder, Hatem Alkadhi, Volkmar Falk, Andreas Boss, Simon P. Hoerstrup

**Affiliations:** 1 Swiss Centre for Regenerative Medicine, University of Zurich, Zurich, Switzerland; 2 Department of Surgical Research, University Hospital of Zurich, Zurich, Switzerland; 3 Clinic for Cardiovascular Surgery, University Hospital of Zurich, Zurich, Switzerland; 4 Institute of Diagnostic Radiology, University Hospital Zurich, Zurich, Switzerland; 5 IMM RECHERCHE, Institute Mutualiste Montsouris, Paris, France; 6 Department of Health Science and Technology, Laboratory for Biologically Oriented Materials, ETH Zurich, Zurich, Switzerland; 7 Department of Pathology, University Hospital of Zurich, Zurich, Switzerland; University of Houston, United States of America

## Abstract

Although stem-cell therapies have been suggested for cardiac-regeneration after myocardial-infarction (MI), key-questions regarding the in-vivo cell-fate remain unknown. While most available animal-models require immunosuppressive-therapy when applying human cells, the fetal-sheep being pre-immune until day 75 of gestation has been proposed for the in-vivo tracking of human cells after intra-peritoneal transplantation. We introduce a novel intra-uterine myocardial-infarction model to track human mesenchymal stem cells after direct intra-myocardial transplantation into the pre-immune fetal-sheep. Thirteen fetal-sheep (gestation age: 70–75 days) were included. Ten animals either received an intra-uterine induction of MI only (n = 4) or MI+intra-myocardial injection (IMI;n = 6) using micron-sized, iron-oxide (MPIO) labeled human mesenchymal stem cells either derived from the adipose-tissue (ATMSCs;n = 3) or the bone-marrow (BMMSCs;n = 3). Three animals received an intra-peritoneal injection (IPI;n = 3; ATMSCs;n = 2/BMMSCs;n = 1). All procedures were performed successfully and follow-up was 7–9 days. To assess human cell-fate, multimodal cell-tracking was performed via MRI and/or Micro-CT, Flow-Cytometry, PCR and immunohistochemistry. After IMI, MRI displayed an estimated amount of 1×10^5^–5×10^5^ human cells within ventricular-wall corresponding to the injection-sites which was further confirmed on Micro-CT. PCR and IHC verified intra-myocardial presence via detection of human-specific β-2-microglobulin, MHC-1, ALU-Sequence and anti-FITC targeting the fluorochrome-labeled part of the MPIOs. The cells appeared viable, integrated and were found in clusters or in the interstitial-spaces. Flow-Cytometry confirmed intra-myocardial presence, and showed further distribution within the spleen, lungs, kidneys and brain. Following IPI, MRI indicated the cells within the intra-peritoneal-cavity involving the liver and kidneys. Flow-Cytometry detected the cells within spleen, lungs, kidneys, thymus, bone-marrow and intra-peritoneal lavage, but not within the heart. For the first time we demonstrate the feasibility of intra-uterine, intra-myocardial stem-cell transplantation into the pre-immune fetal-sheep after MI. Utilizing cell-tracking strategies comprising advanced imaging-technologies and in-vitro tracking-tools, this novel model may serve as a unique platform to assess human cell-fate after intra-myocardial transplantation without the necessity of immunosuppressive-therapy.

## Introduction

Stem cells have been repeatedly suggested as a next generation therapeutic approach for the treatment of heart failure due to myocardial infarction or cardiomyopathy [1]. Based on various animal trials, there are increasing numbers of early phase patient trials that aim to demonstrate the feasibility and potential efficacy of stem cell-based therapies in the clinical setting [2]–[6]. However, despite the plethora of generated data in the field [7], the in-vivo cell fate with specific regards to cell retention and engraftment, survival, and importantly contribution to cardiac regeneration after stem-cell transplantation into the heart remains to be elucidated. One major reason is certainly the too rapid translation from small animal studies or non-comparable large animal studies (mainly pigs and sheep) to clinical human studies, while only a systematic evaluation of the early and late stem cell fate will allow defining the optimal stem cell therapy concept for sustained cardiac regeneration.

To assess the cell fate including cellular in-vivo bio-distribution, engraftment and survival after transplantation, a surrogate animal model is mandatory allowing for sufficient cell tracking in absence of any immunologic rejection [8]–[11]. However, with the exception of gene-modified murine models, the availability of suitable animal models to assess human stem cell fate and bio-distribution is very limited. As most available animal models are associated with the necessity for immunosuppressive therapy when applying human cells, the clinical relevance of findings obtained from such animal models is compromised.

The fetal sheep has been suggested to be an optimal animal model for the assessment of human cell-fate [8]–[15]. Although the fetal sheep has a normal functioning immune-system, it is still able to support human cell engraftment and differentiation if the cells are transplanted before day 75 of gestation [8]–[11], [16]. Following ultrasound-guided, intra-peritoneal stem cell transplantation, previous reports have shown that the fetal sheep is immunologically tolerant to human skin grafts and to allogenic or xenogenic stem cells during this “pre-immune” period of development allowing for a significant engraftment of human cells without the necessity of immunosuppressive therapy [8], [9], [16]–[23]. Taking this unique advantage of this pre-immune status as well as the large size and the long life-span into account, the fetal sheep represents an highly interesting animal model to study human cell-fate offering experimental opportunities that are not available in murine models [10], [11], [16].

In this study and for the first time, we investigated the feasibility to use the pre-immune fetal sheep model for the assessment of human stem cell fate after direct intra-myocardial mesenchymal stem cell transplantation following acute myocardial infarction with specific attention to cell retention and early bio-distribution using advanced, imaging-guided cell tracking protocols.

## Materials and Methods

### Study Animals & Experimental Design

**Figure 1:**
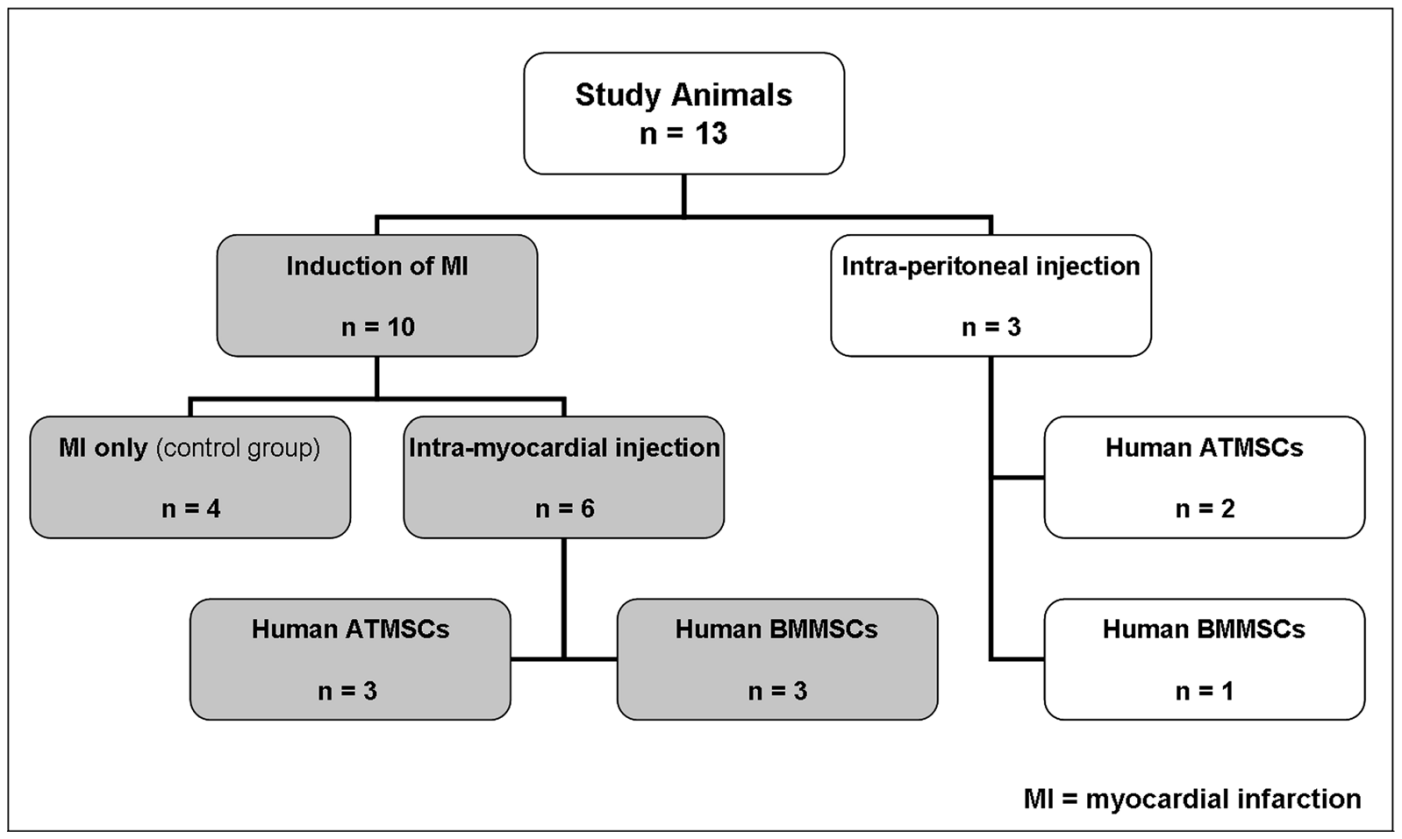
Study Design and Animal Distribution.

We studied 8 pregnant Pre-Alp ewes between 70 to 75 days' gestation (term 145 days) with a total of 13 fetal-sheep. Ten animals either received an intra-uterine induction of MI only (n = 4) or MI + intra-myocardial injection (IMI; n = 6) using micron-sized, iron-oxide (MPIO) labeled human mesenchymal stem cells either derived from the adipose tissue (ATMSCs; n = 3) or the bone-marrow (BMMSCs; n = 3). Three animals received an intra-peritoneal injection (IPI; n = 3; ATMSCs; n = 2/BMMSCs; n = 1) (figure 1). All animals received humane care in compliance with the “Principles of Laboratory Animal Care” as well as the US National Institutes of Health guidelines for the care and use of animals. All procedures were approved by the Institutional Ethics Committee of the IMM RECHERCHE, Institut Mutualiste Montsouris, Paris, France [Approval-No 10-18/2010]. Figure 1 summarizes the study design and the animal distribution.

### Cell Isolation, Characterization and Labeling

#### Isolation of human bone marrow derived mesenchymal stem cells (BMMSCs)

After written informed consent and approval of the institutional review board of the University Hospital of Zurich, 80 mL of bone marrow was aspirated from the sternum of a 52 years old male patient using a bone marrow aspiration syringe with a needle prior to cardiac surgery at the University Hospital Zurich. BMMSCs were isolated as previously described [24]. *Please see supporting file S1 for further details*.

#### Isolation of human adipose tissue derived mesenchymal stem cells (ATMSCs)

ATMSCs (isolated from a 45 years old female patient after written informed consent and approval of the institutional review board of the University Hospital of Zurich) were generously provided by Dr. Maurizio Calcagni from the Clinic for Plastic and Reconstructive Surgery, University Hospital Zurich. The cells were isolated from tissue according to a standard protocol as described elsewhere [25].

#### Labeling of human BMMSCs and ATMSCs with CM-DiI and MPIOs

The cells were first labeled with a fluorescent dye (CellTracker CM-DiI, Invitrogen, Switzerland) according to the manufacturer's instructions. CM-DiI is a lipophilic carbocyanine tracer and incorporates into membranes. Afterwards the cells were incubated with culture medium supplemented with 1.63 um-large encapsulated super-paramagnetic microspheres (MPIOs; Bang Laboratories, USA) at a final concentration of 2.75 ug/ml iron for 24 to 36 hours as described previously [26]. The particles were co-labeled with Dragon-green fluorochromes for additional analysis using fluorescence-based techniques. *Please see supporting file S1 for further details*.

### The Fetal Sheep Cell Transplantation Model

The fetal sheep model has proven to be an appropriate model for the assessment of in-vivo cell fate and bio-distribution of human cells with specific regards to survival and engraftment [10]–[12], [14], [16]. In contrast to other animal models either being genetically modified (i.e. nude mice) or requiring immunosuppressive therapy (xenogenic models such as adult porcine or ovine models) for the assessment of human stem cells, the fetal sheep has a normal functioning immune system, but is still able to support cell engraftment and survival if the cells are transplanted before day 75 of gestation (term 145 days). Taking advantage of this “window of opportunity” and this “pre-immune” period of development, this model allows for a significant engraftment of human cells without the necessity of immunosuppressive therapy [8]–[12], [14], [16].

### Operative Procedures

Following overnight fasting, the animals were sedated by intravenous injection of pentothal (10 mg/kg body weight). The animals were placed in supine position, intubated, ventilated with 100% oxygen and 1–2% isoflurane and monitored continuously throughout the entire procedure. No anti-arrhythmic agents were used during or after the procedure. After the ewes were placed in dorsal recumbency on the operating table, the uterus was exteriorized through a maternal midline laparotomy. Following digital palpation of the fetus, the uterus was opened through a 10 cm incision. The amniotic fluid was collected, stored in a sterile reservoir and out back into the ewe's belly to maintain the temperature until the end of the procedure. While the upper part of the fetal body remained within the maternal uterine cavity, the fetal chest was opened via a left-sided mini-thoracotomy (4^th^ intercostals space). After sharp dissection of the pericardium, the heart was positioned for an optimal access of the anterior wall and the apex.

#### Induction of Myocardial Infarction

After evaluation of the myocardial vasculature, the left anterior descending coronary artery (LAD) and the diagonal branches (DB) were identified. To achieve a sufficient myocardial infarction involving the anterior wall and the apex, the LAD (± appropriate diagonal branches) were suture ligated using a 7/0 suture. Sufficient ligation was confirmed by instant changes of the regional wall movement and the colour in the anterior-apical area.

### In utero Stem Cell Transplantation

#### Intra-myocardial Injection

After ligation of the coronary vessels and an ischemic period of 15–20 min to achieve sufficient anterior-apical myocardial damage, 6 target zones for cell transplantation were defined in the left-ventricular anterior area above the apex. Following careful exposure of the fetal heart, a mean of 1.25±0.30×10^6^ cells was slowly injected (60 sec) into the fetal myocardium using a 29 gauge needle (table 1). The exact injection sites were carefully documented. After stem cell delivery, the absence of myocardial bleeding from the injection sites and cardiac arrhythmia was confirmed before layered closing of the fetal chest using Vycril sutures. Thereafter, the fetus was carefully re-positioned with particular regards to the umbilical cord and the amniotic fluid was re-injected before closing the uterus.

**Table 1:** Preoperative & Procedural Data.

Study Animals	Myocardial Infarction n = 4	MI+Intra-myocardial tem Cell Transplantation = 6	Intra-peritoneal tem Cell Transplantation = 3
Cell Type (ATMSCs/BMMSCs) (n)	n/a	3/3	2/1
Number of Cells (Mio/10^6^)	n/a	1.25±0.30	11.75±2.81
Injection Volume (ul)	n/a	100±30	960±60
Number of Injections (n)	n/a	6	1
Injection Needle (gauge)	n/a	29	23
Duration of Procedure (min)	32±4	51±9	33±11
Waiting Time between MI Induction and Cell Injection (min)	n/a	19±1	n/a
Procedural Mortality (%)	0 (0%)	0 (0%)	0 (0%)
Major procedural complications	none	none	none
Mortality during Follow Up	1 (25%)	2 (33%)*	0 (0%)

* *the two animals that died had received human BMMSCs.*

Animal Weight (gr) / Size (cm): 331 ± 81 / 20 ± 2. Overall Survival (%): 10 (77%).

#### Intra-peritoneal Injection

The procedure was performed as previously described [20], [21], [23]. *Please see supporting file S1 for further details*.

### Post-interventional Care, Follow Up and Animal Harvest

After cell transplantation, the fetal heartbeat was monitored to confirm post-procedural wellbeing. After confirming stable hemodynamic conditions the anaesthetics were stopped. The ewes were left to recover and checked daily for their health status and signs of spontaneous abortion. After 7–9 days post transplantation the ovine fetuses were delivered abdominally. In brief, the harvest procedure was as follows: a general anesthesia of the ewe was initiated with a dose of Sodium Thiopental 10 mg/kg (Nesdonal®, Merial, Lyon, France) which was then maintained with Isoflurane 2% in 100% O_2_ (Attane™ Isoflurane, JD Medical, Phoenix, United States of America) during the surgery. Thereafter, euthanization was of the ewe was performed by an intravenous injection of Pentobarbital (Vétoquinol, Lure, France) and by intracardiac injection of Dolethal (Vétoquinol, Lure, France) in the ovine fetuses.

Next the fetal organs were harvested and processed for further analysis in compliance with the “Principles of Laboratory Animal Care” as well as the US National Institutes of Health guidelines for the care and use of animals. All sacrifice and harvest procedures were approved by the Institutional Ethics Committee of the IMM RECHERCHE, Institut Mutualiste Montsouris, Paris, France [Approval-No 10-18/2010].

### Histology & Immunohistochemistry of myocardial infarction

To assess myocardial infarction, necrosis and cell death, standardized H&E staining, Masson's Trichrome and van Giesson staining was performed. In addition, cleaved Caspase-3 staining (1∶300; Cell Signaling Technology) and TUNEL staining was performed to further access the presence of apoptosis. *Please see supporting file S1 for further details*.

### Detection and tracking of CM-DiI^+^/MPIO^+^ human mesenchymal stem cells in the fetal sheep myocardium

To assess the presence and survival of CM-DiI^+^/MPIO^+^ human mesenchymal stem cells a multimodal evaluation strategy was applied comprising Flow Cytometry, PCR and immunohistochemistry. In brief, the heart was processed as follows: After cutting away the apical and basal region, the left ventricular, anterior region (area of injection) was divided into three parts (each approximately 5 mm×5 mm) which were then either processed freshly for Flow-Cytometry (part 1), snap frozen for PCR (part 2) or in formaldehyde for immunohistochemistry (part 3). *Please see supporting file S1 for further details*.

### Assessment of intrinsic immune response versus CM-DiI^+^/MPIO^+^ human mesenchymal stem cells in the fetal sheep myocardium

To assess a potential intrinsic immune response against the injected CM-DiI^+^/MPIO^+^ human mesenchymal stem cells a detailed immunohistochemical analysis for ovine inflammatory cells was performed using anti-human antibodies reactive to CD3 (Thermo Scientific, Switzerland), CD20 (Cell marque Lifescreen Ltd., Switzerland) and CD68 (Biosystems Switzerland AG, Switzerland) to detect CD3^+^ T-cells, CD20^+^ B-lymphocytes and CD68^+^ macrophages in the fetal myocardial tissue respectively. The detection of ovine inflammatory cells using cross-reactive anti-human antibodies was histologically confirmed on ovine spleen tissue sections.

### Magnetic Resonance Imaging (MRI)

All measurements were performed in a Bruker 4.7T Biospec 47/40 with a gradient strength of 60 mT/m and a slew rate of 1100 T/m/s equipped with a circular polarized 1H mouse whole body RF coil. The imaging protocol consisted after gradient-echo (GRE) localizers in 3 spatial directions of 2D T2w fast spin-echo (FSE) sequences (TR/TE 2500 ms/11 ms; effective TE 33 ms; echo train length 8; matrix 256×256; FoV 40×40 mm; slice thickness 1 mm; averages 3) in transversal, sagittal and coronal orientation.

To estimate the amount of MPIO-labelled cells in the myocardium an in vitro dilution series was performed. Therefore labelled human MSCs of various counts (1×10^6^, 5×10^5^, 1×10^5^, 5×10^4^, 2×10^4^) were mixed in each case with 1×10^6^ ovine cardiomyocytes. The cell pellets were embedded in a 2% (w/v) agarose gel in PBS. The measurement was performed as described above.

### Micro Computed Tomography (Micro CT)

The Micro-CT analysis was performed using a Mirco50 tomographer (Scanco Medical; 90 kVp) with a voxelsize of 1.2 um.

### Disclosures and Freedom of Investigation

The equipment and technology used in the study were purchased using academic funds. The authors had full control of the design of the study, methods used, analysis of data, and production of the written report.

### Statictical Analysis

Quantitative data are presented as mean ± standard deviation (SPSS 17.0, IBM, Somers, NY, USA).

## Results

### Characterization and MPIO Labeling of human BMMSCs and ATMSCs

**Figure 2:**
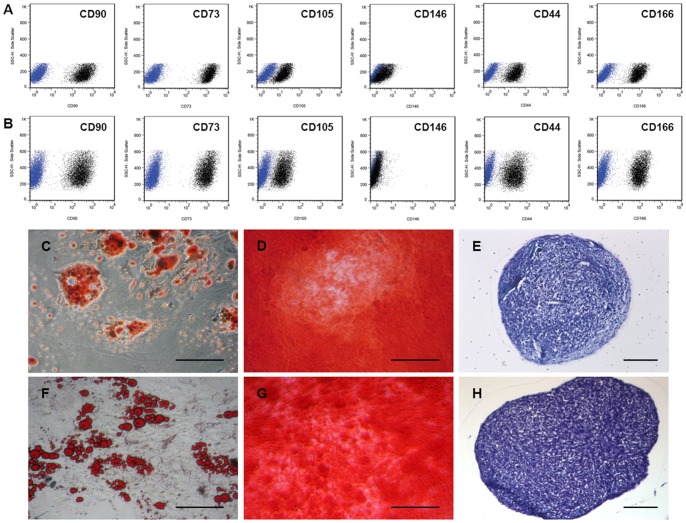
Characterization of human BMMSCs and ATMSCs. Cell surface proteins of human BMMSCs and ATMSCs were evaluated by flow cytometric analysis (**panel A and B**; *blue population represents isotype control*). Positive expression of CD44, CD73, CD90, CD105, CD166 and none/weak expression of CD146 was observed for both MSC cell types. Human BMMSCs (**C–E**) and ATMSCs (**F–H**) demonstrated their differentiation potential to the adipogenic *(Oil Red O Staining, (C, F))*, osteogenic *(Alizarin Red S Staining, (D, G))* and chondrogenic lineages *(Toluidine Blue Staining, (E, H)). Scale Bar: 100 um*.

Cell surface proteins of human BMMSCs and human ATMSCs were evaluated by Flow cytometry. Positive expression of CD44 (mean ± SD, 93.0%±4.3%), CD73 (97.8%±0.3%), CD90 (97.5%±0.7%), CD105 (84.2%±12.5%), and CD166 (94.7%±3.3%) was observed for both MSC cell types, while there was none or only weak expression of CD146 (29.4%±14.7%). Negative levels were detected for CD31 (2.5%±3.5%), CD34 (2.8%±3.9%) and CD45 (2.5%±1.9%) (figure 2A and B). Using the assays described, human ATMSCs and human BMMSCs demonstrated their differentiation potential to the adipogenic, osteogenic and chondrogenic lineages (figure 2C–H).

**Figure 3:**
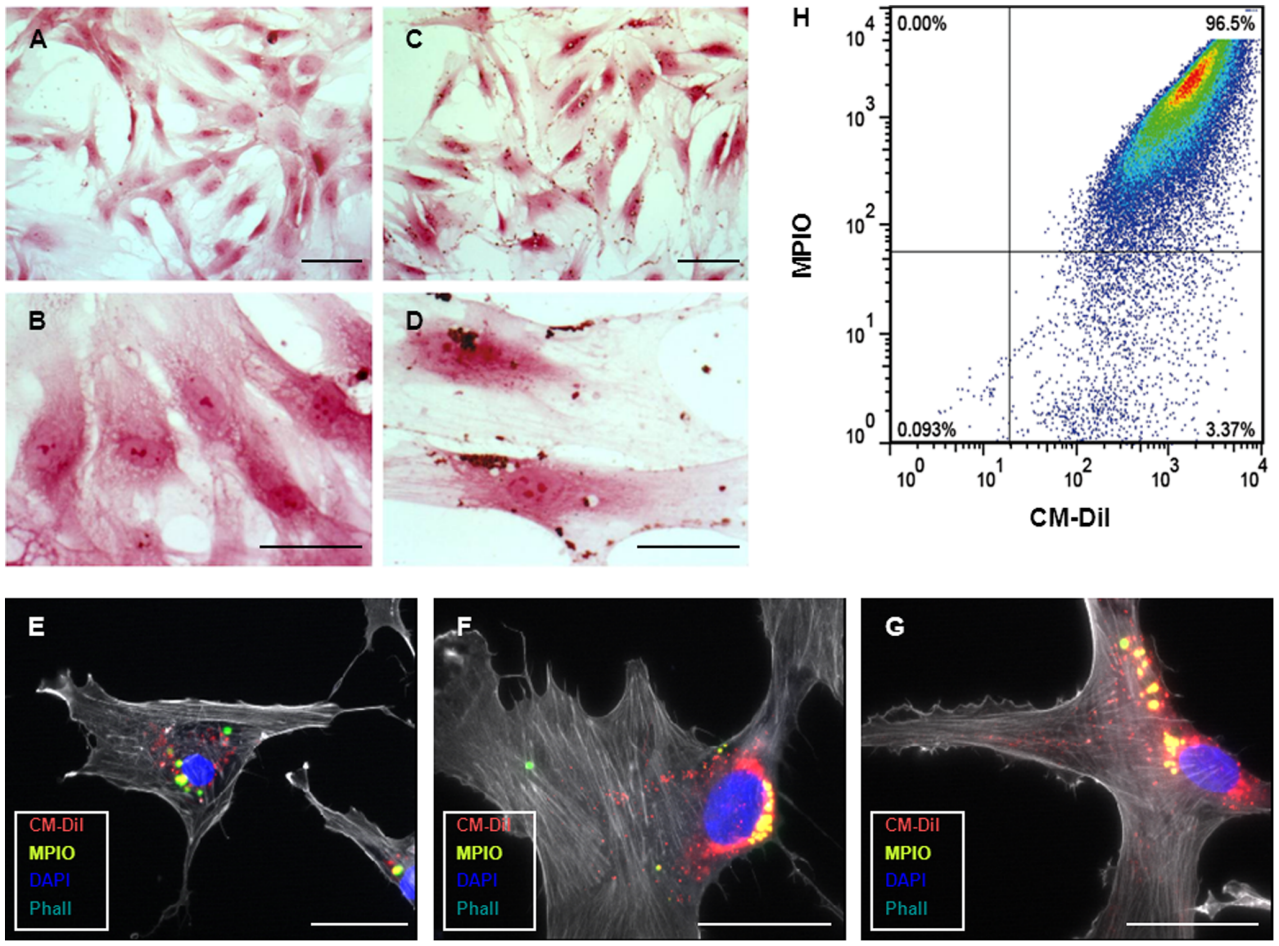
Labeling of human BMMSCs and ATMSCs with superparamagnetic microspheres (MPIOs; co-labeled with Dragon-green fluorochromes) and CM-Dil. Successful labeling of human BMMSCs and ATMSCs was demonstrated on Prussian Blue staining before (**A and B**) and after MPIO labeling (**C and D**) as well as on immunofluorescence clearly showing the presence of MPIOs (**E–G**). After labeling, CM-Dil^+^/MPIO^+^ cells displayed excellent cell labeling efficiency in excess of 95% on FACS analysis (**H**). *Scale Bar: 100 um (A and C), 50 um (B, D–G)*.

Successful labeling of human BMMSCs and human ATMSCs was demonstrated on Prussian Blue staining as well as on immunofluorescence clearly showing the presence of MPIOs (figure 3A–G). This was further confirmed on FACS analysis which also demonstrated an excellent cell labeling efficiency in excess of 95% (figure 3H).

### Surgical Procedures and Induction of myocardial infarction

**Figure 4:**
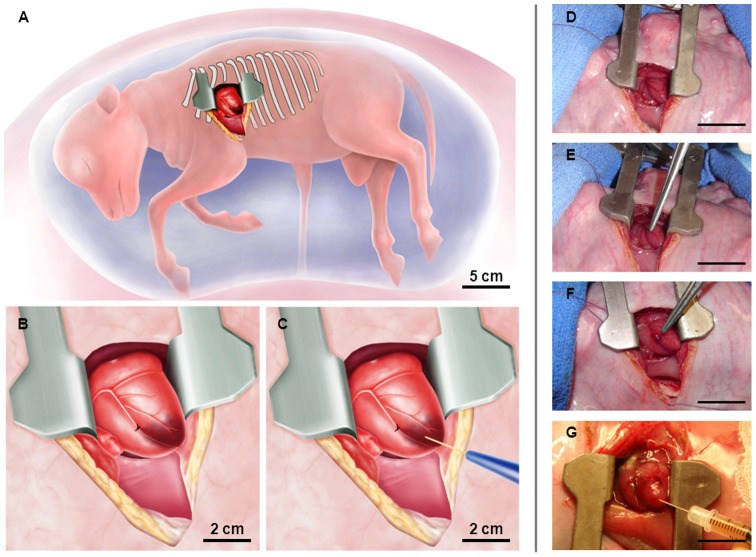
Concept of intra-uterine induction of myocardial infarction and intra-myocardial stem cell delivery into the pre-immune fetal sheep. The uterus was exteriorized through a maternal midline laparotomy. Following digital palpation of the fetus, the uterus was opened through a 10 cm incision. The fetal chest was opened via a left-sided mini-thoracotomy *(4^th^ intercostal space)* (**A**). After sharp dissection of the pericardium, the heart was positioned for an optimal access of the anterior wall and the apex (**A and D**). After evaluation of the myocardial vasculature, the left anterior descending coronary artery (LAD) and the diagonal branches (DB) were identified (**E**). To achieve a sufficient myocardial infarction involving the anterior wall and the apex, the LAD (± appropriate diagonal branches) were suture ligated using a 7/0 suture (**B and F**). Sufficient ligation was confirmed by instant changes of the regional wall movement and the colour in the anterior-apical area (**B and F**). After ligation of the coronary vessels, the 5–6 target zones for stem cell delivery were defined. Following careful exposure of the fetal heart, the cells were slowly injected into the fetal myocardium (**C and G**). *Scale Bar: 5 cm (D–G)*.

**Figure 5:**
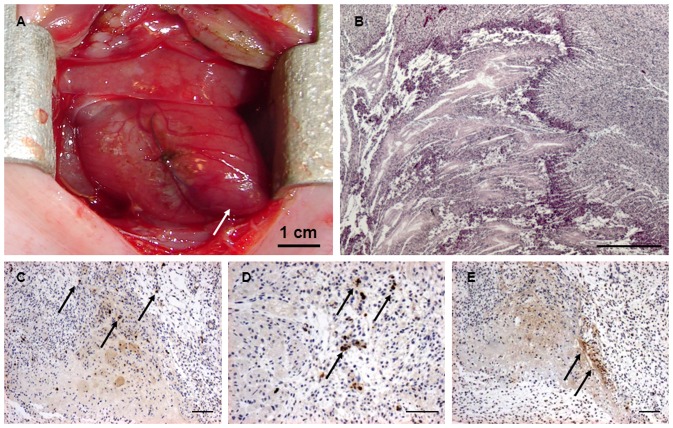
Assessment of intra-uterine induction of myocardial infarction in the pre-immune fetal sheep. Induction of acute myocardial infarction (MI) was confirmed by instant changes of the regional wall movement and the colour in the anterior-apical area (**A**; *purple discolouration; white arrow*). The area of myocardial infarction (MI) could be easily identified showing the typical areas of necrosis when compared to the surrounding border zone and the healthy myocardium. In detail, loss of the classical myocardial morphology, necrotic cell death with the loss of the nuclei and cardiac proteins as well as the complete loss of entire muscle fibres could be observed (**B**; *magnification x2.5*). MI was further confirmed on histology via positive staining for activated caspase-3 (**C and D**; *black arrows*) and TUNEL (**E**; *black arrows*) in fetal infarcts at 7 days suggesting programmed cardiomyocyte death within the infracted region. *Scale Bar: 500 um (B), 100 um (C–E)*.

The surgical procedure and the induction of myocardial infarction (MI) could be successfully performed in all animals (figure 4A–G) scheduled for intra-myocardial stem-cell transplantation or MI only (n = 10, table 1) and was intra-operatively confirmed by instant changes of the regional wall movement and the colour in the anterior-apical area (figure 5A). The respective animals remained hemodynamically stable during the entire procedure and did not display any major complications (table 1). In three animals, a short bradycardic episode was present immediately after ligation of the coronary vessel, but normalized quickly after a few minutes. In addition, two animals encountered mild bleeding from the stitching channels of the ligation suture which self-terminated after a few minutes or was managed with a hemostatic sealant.

The successful induction of myocardial infarction was also confirmed on histology and immunohistochemistry. On H&E staining, the area of myocardial infarction (MI) could be easily identified showing the typical areas of necrosis when compared to the surrounding border zone and the healthy myocardium (figure 5B). In detail, loss of the classical myocardial morphology, necrotic cell death with the loss of the nuclei and cardiac proteins as well as the complete loss of entire muscle fibres could be observed (figure 5B). These findings were further confirmed on Masson's Trichrome and van Giesson staining (data not shown). In addition, also an apoptotic component could be confirmed by positive staining for activated caspase-3 and TUNEL suggesting a programmed cell-death in the area of infarction (figure 5C–E).

#### Intra-myocardial and intra-peritoneal stem cell transplantation

After induction of MI and the definition of 5–6 target zones in the antero-apical area, intra-myocardial transplantation either using human BMMSCs (n = 3) or ATMSCs (n = 3) was successfully performed. All animals tolerated the procedure very well without any major complications. Two animals displayed transient arrhythmia during intra-myocardial stem cell delivery and one animal had minor bleeding from the injection sites.

The intra-peritoneal transplantation was also carried out successfully in all animals (n = 3; ATMSCs; n = 2/BMMSCs; n = 1) and no complications such as bleeding or organ damage occurred (table 1).

### Postoperative Complications and Follow Up

Postoperatively, no major complications occurred in the ewes and in the fetuses. However, as known from previous reports [20], [21] and despite postoperative monitoring, during follow up, three (23%) of the fetuses of which one animal underwent induction of MI only and two animals had received intramyocardial transplantation of BMMSCs died due to spontaneous abortion and were expelled overnight which was primarily related to the fragile in-utero approach itself. Table 1 summarizes the preoperative and procedural data.

### MRI and Micro CT for Stem Cell Tracking

**Figure 6:**
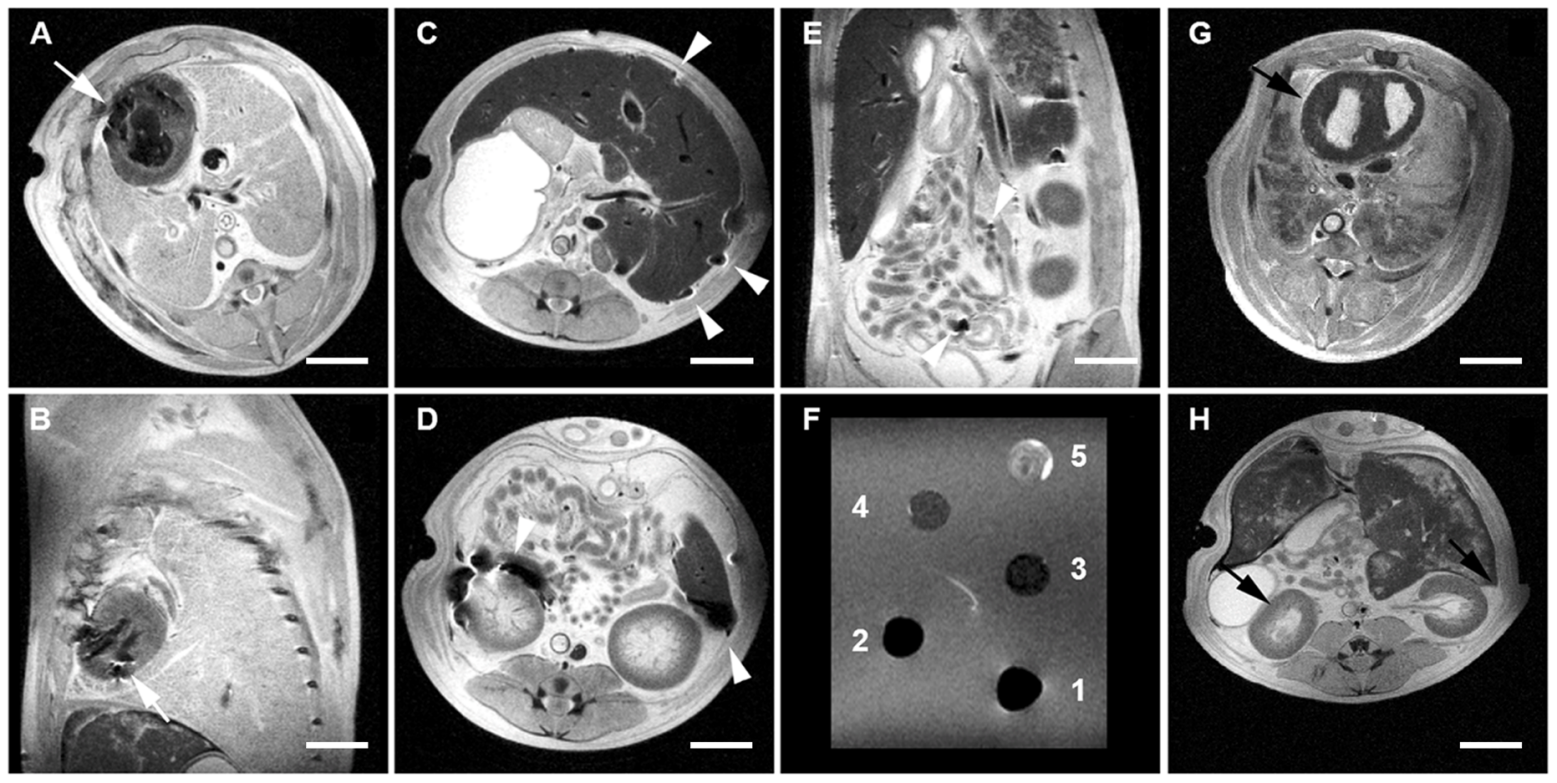
Tracking of CM-DiI^+^/MPIO^+^ human ATMSCs and BMMSCs on two-dimensional magnetic resonance imaging (MRI). CM-DiI^+^/MPIO^+^ human ATMSCs and BMMSCs could be detected after stem cell transplantation on high resolution 4.7 Tesla MRI. Exemplary image series of an animal that had received an intra-myocardial injection of CM-DiI^+^/MPIO^+^ human ATMSCs the labeled cell clusters were clearly visible as areas of strong focal signal loss in the anterior-lateral and septal ventricular wall corresponding to the injection sites (**A and B**; *white arrows; axial and sagittal view*; **and** **G**; *black arrow*; *showing a non-injected myocardium as respective negative control*). Next, an exemplary image series of an animal that underwent intra-peritoneal injection of CM-DiI^+^/MPIO^+^ human BMMSCs displayed the distribution within the entire intra-peritoneal cavity (**C–E**; *white arrow heads*; **and H**; *black arrows*; *showing a non-injected intra-peritoneal cavity as respective negative control*) involving the liver (**C**; *white arrow heads; axial and view*), the kidneys, the Gerota's fascia (**D**; *white arrow heads; axial view*), diffuse cell clusters in the surrounding intra-peritoneal areas (**D**; *white arrow heads; axial view*) as well as between the intestines (**E**; *white arrow heads; sagittal view*). Comparing the dilution series (**F**) of MPIO labeled mesenchymal stem cells to the morphological images, the amount of cells in the myocardium could be estimated to approximately 1×10^5^–5×10^5^ cells and the cells found in the liver to 5×10^5^ cells and around the kidneys to 5×10^5^–1×10^6^ cells (**A–D and F**). *(Cell Dilution: 1×10^6^* *[1]*, *5×10^5^* *[2]*, *1×10^5^* *[3]*, *5×10^4^* *[4]*, *2×10^4^* *[5]* *. Scale Bar: 2.5 cm*.

CM-DiI^+^/MPIO^+^ human BMMSCs and ATMSCs could be successfully detected after stem cell transplantation on high resolution MRI. In selected animals that had received an intra-myocardial transplantation after MI, the labeled cell clusters were clearly visible as areas of strong focal signal loss in the septal and anterior-lateral ventricular wall corresponding to the injection sites (figure 6A and B). In addition, cells were also detectable in the costal area, para-vertebral and in the Sinus Phrenico Costalis indicative that the cells had been distributed in the thorax over time.

Animals that underwent an intra-peritoneal transplantation displayed a distribution within the entire intra-peritoneal cavity involving the liver, the kidneys, the Gerota's Fascia, intestines as well as diffuse cell clusters in the surrounding areas (figure 6C–E).

Comparing the dilution series (figure 6F) of MPIO-labeled cells to the morphological images, the amount of cells in the myocardium could be estimated to approximately 1×10^5^–5×10^5^ cells and the cells found in the liver to 5×10^5^ cells and around the kidneys to 5×10^5^–1×10^6^ cells.

**Figure 7:**
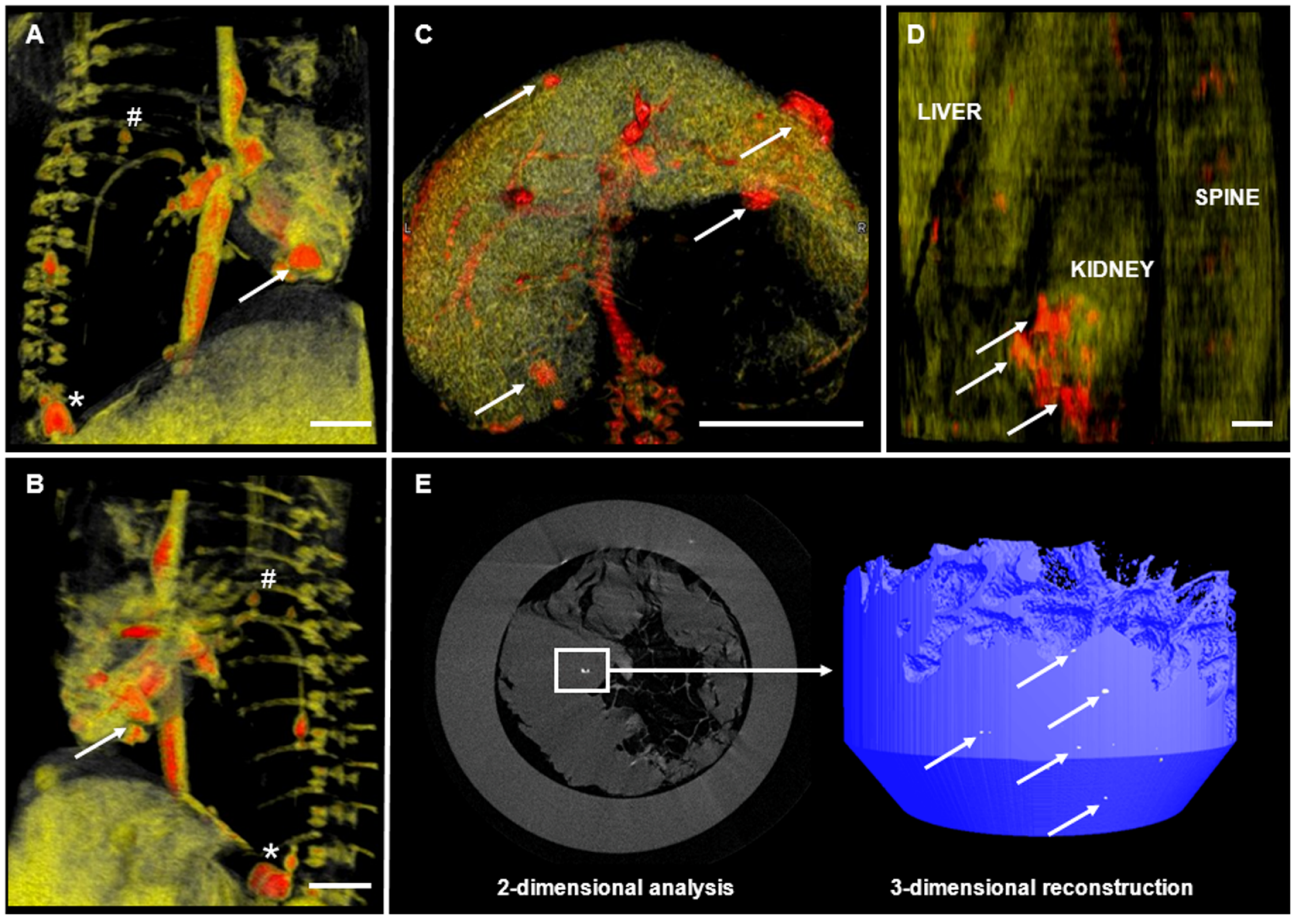
Tracking of CM-DiI^+^/MPIO^+^ human ATMSCs and BMMSCs on three-dimensional reconstruction MRI and Micro CT Scanning. Exemplary three-dimensional reconstruction analysis confirmed labeled cell clusters of human CM-DiI^+^/MPIO^+^ ATMSCs after intramyocardial transplantation in the anterior-lateral and septal ventricular wall (**A and B**; *white arrows; anterior-lateral and septal view*). In addition, labeled cells were also detectable in the costal/para-vertebral area (**A and B**; white *) and in the Sinus phrenico-costalis (**A and B**; *white #*) indicative that the cells had been distributed in the fetal thorax. Abdominal 3D reconstruction analysis showed labeled cell clusters of CM-DiI^+^/MPIO^+^ human BMMSCs following intra-peritoneal injection within and around the liver (**C**; *white arrows; anterior-inferior view*) as well as in the anterior the Gerota's fascia (**D**; *white arrows; lateral view*). The intra-myocardial presence of CM-DiI^+^/MPIO^+^ human ATMSCs could be further confirmed showing several labeled cell clusters in the fetal myocardium on 2-dimensional as well as 3-dimensional high-resolution Micro CT analysis (**E**). *Scale Bar: 2 cm*.

Three dimensional MRI reconstruction confirmed the previous results on 2D MRI (figure 7A–D) and in selected tissue samples, the intra-myocardial presence of the iron-oxide labeled cells was further confirmed high-resolution Micro CT showing several labeled cell clusters in the myocardium (figure 7E).

### Explant Macroscopy

Ten fetuses completed follow-up and could be uneventfully harvested 7–9 days post transplantation prior to ewes euthanization. The fetal heart was carefully excised and the ligation site as well as the area of myocardial infarction could be easily identified. In selected animals, the heart appeared to be slightly adhered to the pericardium and required careful dissection. The other fetal organs including lungs, liver, kidneys, spleen and others also appeared to be in good shape and were harvested for further analysis. Interestingly, most of the animals that had received left-sided thoracotomy displayed almost complete healing of the incision already after 7–9 days indicating an accelerated healing and regeneration potential in the fetal stage.

### Assessment of cell fate and early bio-distribution of injected human BMMSCs and ATMSCs

#### Detection of CM-DiI^+^/MPIO^+^ human ATMSCs and BMMSCs in fetal tissue by Flow Cytometry

**Figure 8:**
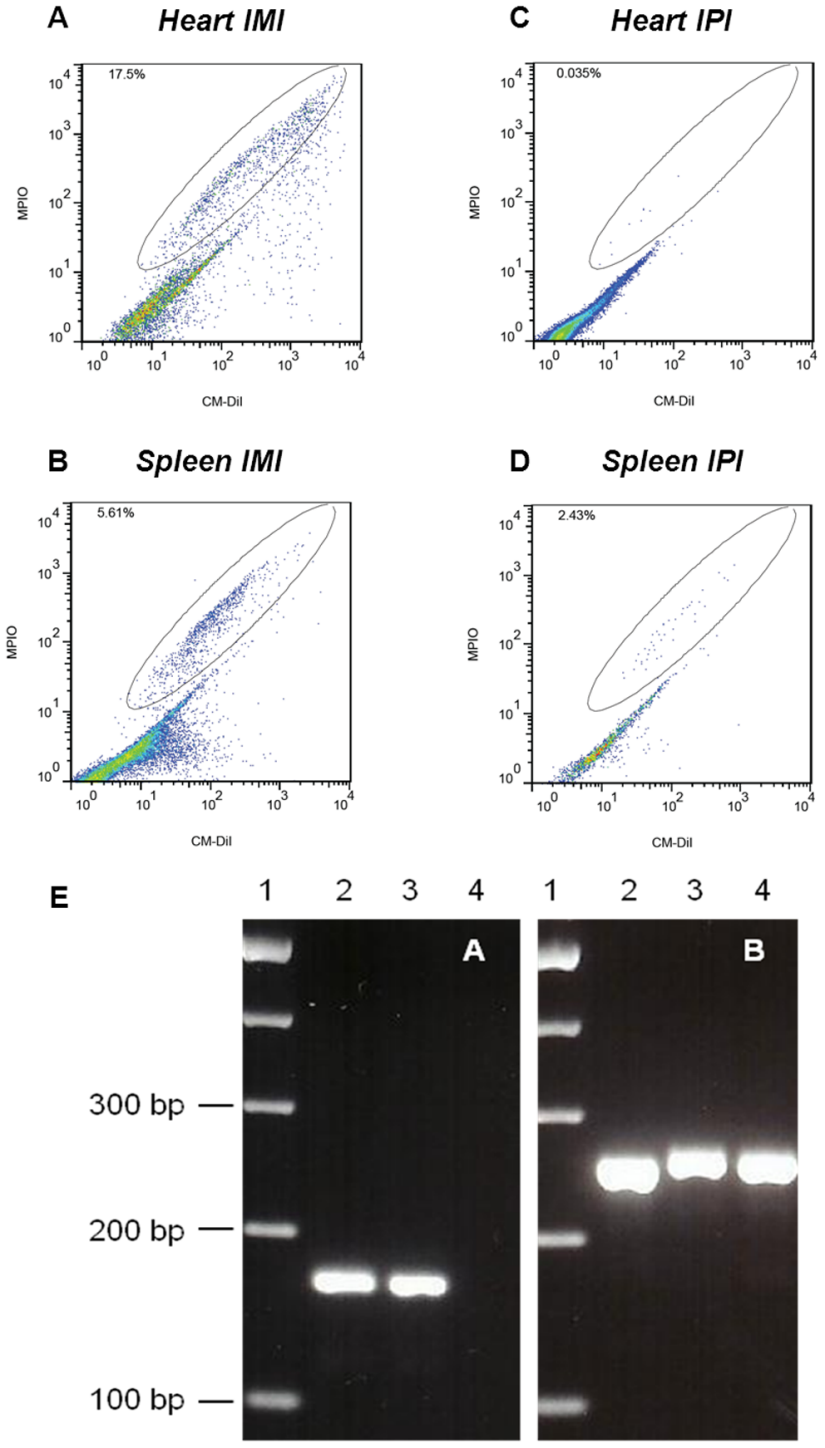
Assessment of cell fate and early bio-distribution of injected of human ATMSCs and BMMSCs via Flow Cytometry and PCR analysis. Flow cytometric analysis in an exemplary recipient after direct intra-myocardial injection (IMI) of human ATMSCs. CM-DiI^+^/MPIO^+^ human ATMSCs were primarily detected within the heart and within the spleen (**A and B**). In an animal that had received intra-peritoneal injection (IPI) of human BMMSCs, CM-DiI^+^/MPIO^+^ BMMSCs were primarily identified within the lymphatic organs, in particular within spleen, while no CM-DiI^+^/MPIO^+^ cells were found within the heart (**C and D**). Intra-myocardial presence of CM-DiI^+^/MPIO^+^ human ATMSCs was further confirmed via PCR analysis using human β-2 microglobulin (**E**), a component of the class I antigen complex. Negative controls from non-injected sheep hearts as well as human mesenchymal stem cells used as a positive control clearly confirmed the human specificity of the staining for human β-2 microglobulin within the fetal heart: Agarose gel analysis of human β-2 microglobulin *(left panel, A)* and β -actin *(right panel, B)* PCR products. Lane 1: molecular size marker (100 bp DNA ladder, Genecraft, Germany). Lane 2: human mesenchymal stem cells as a positive control. Lane 3: ovine fetal heart after human ATMSCs injection. Lane 4: tissue from sheep heart as a negative control for the human β-2 microglobulin sequence.

After intra-myocardial injection, presence of CM-DiI^+^/MPIO^+^ human ATMSCs and BMMSCs could be primarily confirmed within the heart and within the spleen (table 2, figure 8 A and B). Positive cells were also found within the bone marrow, the kidneys, lungs and in the brain (table 2).

**Table 2:** Distribution of CM-DiI^+^/MPIO^+^ human mesenchymal stem cells in ovine fetal tissue by Flow Cytometry.

	Intra-myocardial Stem Cell Transplantation		Intra-peritoneal Stem Cell Transplantation	
Tissue	Analyzed	Detected	Analyzed	Detected
	n	n (%)	n	n (%)
Heart	4	4 (100%)	3	0 (0%)
Brain	4	3 (75%)	3	0 (0%)
Spleen	4	4 (100%)	3	2 (66%)
Kidneys	4	4 (100%)	3	2 (66%)
Lavage	4	0 (0%)	3	3 (100%)
Bone Marrow	2	2 (100%)	3	2 (66%)
Liver	4	1 (25%)	3	1 (33%)
Lung	4	3 (75%)	3	2 (66%)
Thymus	4	1 (25%)	2	2 (100%)

In animals that had received intra-peritoneal injection, CM-DiI^+^/MPIO^+^ human ATMSCs and BMMSCs were primarily identified within the lymphatic organs, including spleen, thymus and the bone-marrow, while in none of the animals CM-DiI^+^/MPIO^+^ cells were found within the heart (table 2, figure 8C and D). Positive cells were also detected in the intra-peritoneal lavage supporting the findings on the MRI analysis. In addition, low levels of positive cells were distributed in different organs, including the liver, lungs and kidneys (table 2).

#### Detection and tracking of CM-DiI^+^/MPIO^+^ human ATMSCs and BMMSCs in the pre-immune fetal sheep myocardium via PCR Analysis and IHC

Following detection of CM-DiI^+^/MPIO^+^ human ATMSCs and BMMSCs in the fetal myocardium in the imaging analysis (MRI and Micro CT scanning) and the FACS assessment, intra-myocardial presence was further confirmed via PCR analysis using human β-2 microglobulin, a component of the class I antigen complex (figure 8E). Negative controls from non-injected sheep hearts as well as human mesenchymal stem cells used as a positive control clearly confirmed the human specificity of the staining for human β-2 microglobulin within the fetal myocardium (figure 8E).

**Figure 9:**
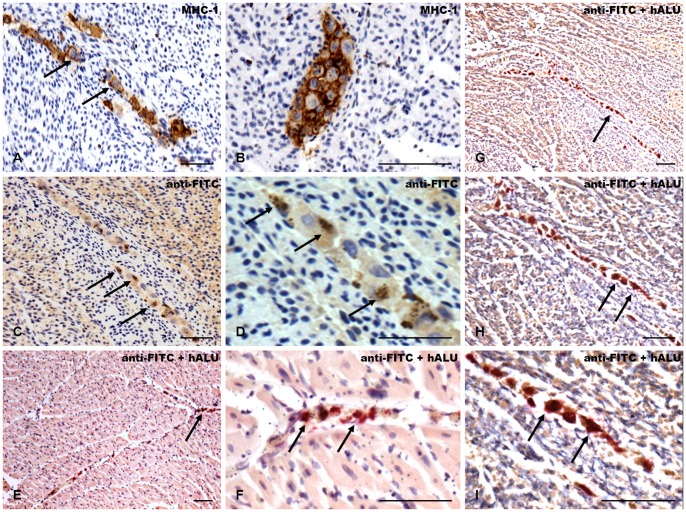
Detection of CM-DiI^+^/MPIO^+^ human ATMSCs in the pre-immune fetal sheep myocardium via immunohistochemistry. Exemplary image series post intramyocardial transplantation of human ATMSCs into the fetal myocardium. Morphologically CM-DiI^+^/MPIO^+^ human ATMSCs could be easily identified within the fetal heart tissue suggesting to be in good shape and viable. The cells appeared to be integrated within the fetal myocardium and could be found as clusters as well as in the interstitial and intravascular spaces (**A–I**; *black arrows*). The cells stained positive for human specific Major Histo Compatibility Complex 1 (MHC-1) clearly confirming the human origin (**A and B**; *black arrows*) and also stained positive for anti-FITC detecting the Dragon Green fluorochrome labelled MPIOs within the human cells (**C and D**; *black arrows*). In addition, double staining for ALU Sequence and anti-FITC further confirmed the presence of the injected CM-DiI^+^/MPIO^+^ human ATMSCs within healthy heart tissue (**E and F**; *black arrows*) as well as in infarcted myocardium (**G–I**; *black arrows) Scale Bar: 100 um (A–C, E, G, H), 50 um (D, F, I)*.

Morphologically the cells could be easily identified within the fetal myocardium, appeared to in physiological shape and viable (figure 9A–I). The cells had integrated within the fetal heart and could be found in clusters as well as in the interstitial and intravascular spaces (figure 9A–I). CM-DiI^+^/MPIO^+^ human ATMSCs and BMMSCs were positive for both human-cell specific MHC-1 staining (figure 9A and B) as well as anti-FITC staining detecting the MPIOs within the cytoplasma of the injected human cells (figure 9C and D). In addition, double positive staining for the human-specific ALU Sequence in combination with the anti-FITC staining clearly confirmed the presence of the injected CM-DiI^+^/MPIO^+^ human ATMSCs and BMMSCs within the fetal myocardium (figure 9E–I).

### Assessment of intrinsic immune response versus CM-DiI^+^/MPIO^+^ human ATMSCs and BMMSCs in the fetal sheep myocardium

**Figure 10:**
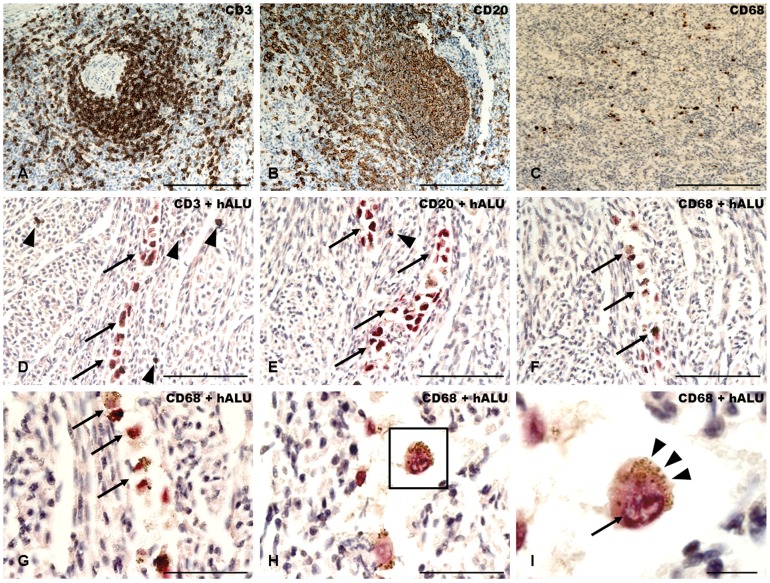
Assessment of intrinsic immune response versus CM-DiI^+^/MPIO^+^ human ATMSCs in the pre-immune fetal sheep myocardium. Control staining of CD3^+^ T-cells, CD20^+^ B-lymphocytes and CD68^+^ macrophages in ovine spleen tissue (**A–C**) After direct intra-myocardial injection of CM-DiI^+^/MPIO^+^ human ATMSCs (**D–I**; black arrows), CD3^+^ T-cells (**D**; black arrow heads) CD20^+^ B-lymphocytes (**E**; black arrow head) could be detected in normal, tissue-specific frequencies and appeared to be disseminated within the entire fetal myocardium without any sign of activation due to an immune response against the human cell graft. In particular, neither a T- or B-cell infiltration into the area of the human cell graft, nor increased numbers of CD68^+^ macrophages (**F**) in this area could be observed. In contrast, CM-DiI^+^/MPIO^+^ ATMSCs could be easily identified morphologically and by positive staining for human-specific ALU-sequence (**D–I**; black arrows). The human cells were in physiological shape (**G and H**; black arrows and black frame) have kept the MPIOs intra-cellular (**I**; black arrow heads) and have not been phagocytized by ovine macrophages. *Scale Bar: 100 um (A–F), 50 um (G and H) and 20 um (I)*.

Detailed assessment for ovine CD3^+^ T-cells, CD20^+^ B-lymphocytes as well as CD68^+^ macrophages in the fetal myocardial tissue did not show a potential intrinsic immune response against the injected CM-DiI^+^/MPIO^+^ human ATMSCs and BMMSCs (figure 10A–I). The ovine immune cells could be detected in normal, tissue-specific frequencies and appeared to be disseminated within the entire fetal myocardium without any sign of activation due to an immune response against the human cell graft (figure 10D–F). In particular, neither a T- or B-cell infiltration into the area of the human cell graft, nor increased numbers of macrophages in this area could be observed (figure 10D–F). In contrast, CM-DiI^+^/MPIO^+^ human ATMSCs and BMMSCs could be easily identified morphologically, and by positive staining for human-specific ALU-sequence as well as the specific intracellular, brown colour dots given by the iron-oxide particles clearly indicating that the human cells were in physiological shape, have kept the MPIOs intra-cellular and have not been phagocytized by ovine macrophages (figure 10G–I).

## Discussion

Stem cells have shown great promise as a therapeutic strategy for the failing heart after myocardial infarction and based on encouraging preclinical studies [7], there are growing numbers of clinical pilot trials showing the principal feasibility of stem cell-based therapies [2]–[6]. However, the rapid and often too premature translation into the clinical arena has left many key questions unanswered with special regards to the in-vivo cell fate which is fundamental to fully understand the possible beneficial effect on the diseased heart. In addition, the availability of suitable animal-models to assess human stem-cell fate and bio-distribution in-vivo is limited. As most available large animal models require immunosuppressive therapy when applying human cells to the myocardium [7] the clinical relevance is compromised and a surrogate animal model is mandatory to evaluate human stem-cell fate including bio-distribution, engraftment and survival in the absence of any xenogenic immune response.

To overcome these limitations, for the first time we demonstrate the principal feasibility to use a pre-immune fetal sheep intra-uterine myocardial infarction model for the assessment of human stem-cell fate after direct intra-myocardial stem-cell transplantation. Using advanced, imaging-guided cell-tracking protocols comprising magnetic-resonance imaging (MRI) and micro computed-tomography (Micro CT) as well as in-vitro cell-tracking tools, this novel model offers an excellent platform to evaluate human cell-fate in the absence of immunosuppressive therapy.

The pre-immune fetal sheep model has been previously suggested to represent an appropriate animal model for the assessment of human cell-fate. Before day 75 of gestation the immune-system of the fetal sheep is normal functioning, but is still largely immuno-naïve supporting the engraftment and differentiation of human stem cells without the necessity of immunosuppressive therapy that would also compromise the transplanted cells [8]–[12], [16], [17]. In line with that, in our study we did not detect a potential intrinsic xenogenic immune response after direct intra-myocardial injection of human mesenchymal stem cells.

While the pre-immune fetal sheep has been primarily used in the field of experimental haematology to assess in-vivo cell-fate and bio-distribution after ultrasound-guided, intra-peritoneal stem cell delivery [9], [20]–[23], [27], this is the first report demonstrating the feasibility of direct intra-myocardial stem-cell transplantation after myocardial infarction. While previous studies reported a limited homing to the myocardium after intra-peritoneal stem-cell transplantation [9], [20]–[23], [27] indicating the clear limitations of this model with regards to myocardial regeneration, our novel concept of intra-uterine, direct intra-myocardial stem-cell transplantation appears to be more appropriate in the setting of cardiovascular stem cell therapy concepts and offers a significant improvement with regards to myocardial cell retention.

In this study, we used an advanced concept of imaging-guided tracking methodology including MRI/Micro-CT Scanning as well as multiple analysis tools comprising Flow Cytometry, PCR and IHC to track human cells in the fetal sheep myocardium. Using novel micron-sized, Dragon-green fluorochrome labelled iron-oxide particles (MPIO) that had so far been used in the field of liver research [28], [29], we were able to evaluate human cell-fate after intra-myocardial stem cell transplantation on MRI which was then followed by detailed Flow Cytometry, PCR and IHC assessment highlighting the advantage of the fluorochrome co-labelled MPIOs. On MRI, the MPIO labeled cell-clusters were clearly visible within the septal and anterior-lateral ventricular-wall corresponding to the injection sites. Thereafter, the intra-myocardial presence was verified on Flow-Cytometry and PCR, before it was further confirmed on IHC via positive staining for human-specific MHC-1, ALU-Sequence as well as anti-FITC detecting the fluorochrome labeled part of the iron-oxide particles. Considering the initially performed dilution series, the amount of cells within the myocardium could be estimated to approximately 1×10^5^–5×10^5^ providing a brief quantitative estimation of cell-retention after intra-myocardial stem-cell transplantation as a precondition to explain potential beneficial effects of stem-cell therapy concepts.

Advanced imaging technologies for stem-cell tracking with regard to survival, engraftment and differentiation represent a key-requisite to validate functional effects of cell-based therapy concepts [30], [31]. Various imaging-concepts are currently under evaluation including MRI imaging with direct cell-labelling of cells using super-paramagnetic agents, PET- or SPECT-imaging using radio-nuclides, as well as reporter-genes [30], [31]. While each of these approaches has advantages and disadvantages, most of the recent large animal-studies applied MRI technology to detect super-paramagnetic agents as this technique has become an important endpoint to demonstrate efficacy in clinical pilot studies [30]. It offers detailed morphologic as well as functional cardiac information and therefore appears to be an appropriate imaging tool to comprise both, efficacy evaluation and the capability for cell-tracking. Current studies aim on refining contrast-agents to achieve a maximum signal for minimum labelling [30]. While the lowest cell amount detectable with a conventional MRI scanner was 10^5^, a recent study highlighted that this threshold of detection can be further reduced whit the use of high resolution scanners (11.7-T) that may even allow for single cell tracking [32]. In addition, preclinical animal studies applying micron- or nano-sized iron-oxide particles showed the feasibility of non-toxic labelling of mesenchymal stem cells (MSCs) without compromising their trans-differentiation capacity [32]–[35].

On the other hand, it has been described that the main disadvantage of using super-paramagnetic agents is the fact that the imaging signal is not directly linked to cell-viability and the inability to discriminate between vital, labelled cells and particle-loaded cell-debris or hosting macrophages that may have actively phagocytised the particles after cell-death significantly confounding a sufficient call tracking analysis [31]. However, utilizing a detailed in-vitro tissue evaluation and importantly, advanced super-paramagnetic agents such as micron-sized, Dragon-green fluorochrome co-labeled iron-oxide particles [28], [29], [36], we were able to proof the MRI findings and to confirm the presence of the transplanted cells within the fetal myocardium. Using specific antibodies to either detect human cells, the fluorochrome co-labeled MPIOs or both, we were able to clearly detect the transplanted cells highlighting the efficacy of this cell-tracking approach.

Finally, while various stem cell sources are currently under evaluation for their ability to promote cardiac repair [37]–[44], in this study we used human mesenchymal stem cells (MSCs) either derived from the bone-marrow or from the adipose tissue as they are considered clinically safe, easily available and immuno-privileged [45]. In particular, bone-marrow derived MSCs representing a benchmark cell for cardiovascular stem cell therapies have been repeatedly used in preclinical animal models [46]–[49] as well as in clinical pilot trials [2], [5], [45]. Importantly, beside their suggested function through multiple paracrine effects [2] recent reports indicate that bone-marrow MSCs can be programmed into a cardiac committed stage increasing their clinical relevance and potential [50]. Similarly, the adipose tissue also represents a rich source of MSCs being well comparable to those derived from the bone marrow [51] with regards to the surface marker profile and the differentiation capacity. Furthermore, due to their abundant availability and the even lesser invasive access, adipose tissue derived MSCs should be considered as an attractive and clinically highly relevant stem cell source for future therapy concepts [52].

There are several limitations in our report that need to be addressed in further studies: First, since it was the overall aim of this proof-of-concept study to establish a novel model of intra-uterine induction of MI and direct intra-myocardial stem cell transplantation in a technically challenging and very delicate fetal environment, the number of animals was low and the follow-up time was relatively short. Therefore, further studies with an increased number of animals and a longer follow-up are mandatory in order to address the important aspect of long-term engraftment and bio-distribution. Secondly, in the context of the presented multimodal approach for an advanced cell tracking comprising Flow-Cytometry, PCR and IHC, it is to mention that the Flow Cytometry results may have been influenced by the location and size of the harvested cardiac sample as well as the gating strategy. To address the key aspect of cellular retention more accurately including absolute number of cells, the analysis of the entire heart with only a single assessment strategy either using Flow-Cytometry or qPCR would be necessary. Third, in this study two different types of human mesenchymal stem cells were used potentially influencing the study results to some extent. In addition, and although the cardiovascular differentiation potential of human mesenchymal stem cells is of particular interest, this was beyond of this proof-of-concept study. Moreover, besides the infarction histology, a functional assessment (i.e. with echo) of the fetal heart after myocardial infarction was not performed in this first feasibility study. Finally, the MRI analysis was based on T2w sequences. To further enhance cell number estimation accuracy, T2w and T2*w relaxation curves of the tissue will be performed.

## Conclusions

For the first time we demonstrate the principal feasibility of direct intra-myocardial stem-cell transplantation following intra-uterine induction of myocardial infarction in the pre-immune fetal sheep. Using an advanced cell-tracking strategy comprising *state-of-the-art* imaging techniques including magnetic-resonance imaging (MRI) and micro computed-tomography (Micro CT) as well as multimodal in-vitro tracking tools, this model offers a unique platform to evaluate human cell-fate and to track human stem cells in a relevant large animal-model without the necessity of immunosuppressive therapy.

## Supporting Information

File S1 **provides further details on materials and methods.**
(DOC)
