# Supplementary material for: Intramyocardial Transplantation and Tracking of Human Mesenchymal Stem Cells in a Novel Intra-Uterine Pre-Immune Fetal Sheep Myocardial Infarction Model: A Proof of Concept Study
Source: PLoS One. 2013 Mar 22;8(3):e57759. doi: 10.1371/journal.pone.0057759 (PMC3606388; doi:10.1371/journal.pone.0057759)
Supplement: File S1 — provides further details on materials and methods. (DOC) [file pone.0057759.s001.doc]

**Supporting File S1:**

**Emmert et al:**

**Intramyocardial transplantation and tracking of human mesenchymal stem cells in a novel intra-uterine pre-immune fetal sheep myocardial infarction model: A proof of concept study**

**Supplementary Materials and Methods:**

**Cell Isolation, Characterization and Labeling**

*Isolation of human bone marrow derived mesenchymal stem cells (BMMSCs)*

Briefly, BM was diluted with Hank’s balanced salt solution (HBSS; Kantonsapotheke Zürich, Switzerland) and layered on a Histopaque 1077-gradient (Sigma Aldrich, Buchs, Switzerland). Mononuclear cells were isolated, washed twice in HBSS and re-suspended in Minimum Essential Medium (MEM) alpha supplemented with 2 mM L-glutamine, 100 U/ml penicillin, 100 ug/ml streptomycin (all Invitrogen, Carlsbad, CA, USA) and 10% (v/v) fetal bovine serum (Biowest, France). The MSCs were cultured in control medium at 37°C in a humidified atmosphere containing 5% CO_2_. Non-adherent cells were removed after 24 hours and the medium was replaced. At near confluency, the cells were detached with 0.05% trypsin-EDTA (Invitrogen, Carlsbad, CA, USA) and then passaged for further expansion and analysis.

*Characterization of human BMMSCs and ATMSCs using Flow Cytometry*

The surface markers of hBMMSCs and hATMSCs were analyzed using the following mouse anti-human monoclonal antibodies: CD31, CD34, CD44 (ImmunoTools, Germany), CD45, CD90, CD105, CD146 (R&D Systems, Switzerland), CD73 and CD166 (BioLegend, Switzerland). Specific expression was determined by comparison with the appropriate isotype control. Cells were incubated with direct labelled primary antibodies for 20 min at 4°C. Finally, the cells were washed with FACS buffer consisting of 2% FCS (Biowest, France), 0.5 M EDTA and 0.05% sodium azide (Sigma Aldrich, Switzerland) in phosphate buffered saline (Invitrogen, Switzerland) and fixed in PBS containing 1% paraformaldehyde (Sigma Aldrich, Switzerland). The samples were acquired by BD FACS Calibur Cytometer (Becton Dickinson, USA) and analyzed using FlowJo software (Tree Star, USA).

*Differentiation Potential of human BMMSCs and ATMSCs*

Expanded hBMMSCs and hATMSCs were characterized for differentiation toward osteogenic, adipogenic and chondrogenic lineages as previously described . Briefly, to induce mineralization, the hBMMSCs were incubated with DMEM low glucose (Invitrogen, Switzerland), 10% FCS (Biowest, France), 10 mM β-Glycerophosphate, 50 µM L-Ascorbic acid 2-phosphate, 10 nM Dexamethasone (Sigma Aldrich, Switzerland) and 1% Penicillin and Streptomycin (Invitrogen, Switzerland). For the differentiation of hATMSCs 100 nM Dexamethasone was used. After three weeks cells were fixed with 4% formalin (Kantonsapotheke Zürich, Switzerland) and calcium deposits were detected using 2% Alizarin red (Sigma Aldrich, Switzerland). For adipogenic differentiation cells were stimulated with induction medium containing DMEM low glucose, 10% FCS, 1 µM Dexamethasone, 10 µg/ml Insulin, 120 µM Indomethacin, 0.5 mM 3-Isobutyl-1-methylxantine (IBMX, Sigma Aldrich, Switzerland) and 1% Penicillin and Streptomycin for 3 days. Afterwards the medium was changed to maintenance medium consisting of induction medium without IBMX supplementation. Three weeks later, the cultures were fixed with 4% formalin and accumulation of lipid vacuoles was stained with 3% Oil Red-O (Sigma Aldrich, Switzerland). Cultures were observed under an inverted microscope (Axiovert 40 CFL, Zeiss, Germany). To evaluate the chondrogenic potential cell aggregates were incubated in induction medium consisting of DMEM high glucose (Invitrogen, Switzerland), 1% FCS, 0.5 µg/ml Insulin, 50 µM L-Ascorbic acid 2-phosphate, 10 ng/ml Transforming growth factor-beta1 (PeproTech, UK) and 1% Penicillin and Streptomycin. After three weeks the pellet was fixed in 4% formalin, paraffin embedded and the staining of the proteoglycan synthesis was performed using 0.1% Toluidine blue (Sigma Aldrich, Switzerland). The slides were analyzed by microscopy (Leica CTR6000, Leica Microsystems AG, Switzerland).

*Labeling of human BMMSCs and ATMSCs with CM-DiI and MPIOs*

To remove excessive MPIOs after labeling, the cells were washed three times with phosphate-buffered saline (PBS; Invitrogen, Switzerland). The viability and labeling efficiency was determined by trypan blue exclusion and FACS analysis, respectively*.* Prussian blue staining was used to detect ferric iron of labelled cells with MPIOs . Cells were fixed with 4% formalin for 1 h and afterwards incubated with Prussian blue staining solution (Accustain, Sigma Aldrich, Switzerland) according to the manufacturer’s instructions. The slides were analyzed with a light microscope (Leica CTR6000, Leica Microsystems AG, Switzerland). Hemosiderin, some oxides and salts of iron are shown in blue up to brown and nuclei and cytoplasm in pink to red.

**In utero, intra-peritoneal Stem Cell Transplantation**

Using trans-uterine ultrasound, the number, positioning, viability and the gestational age of the fetuses (measuring crown-rump length) was determined. Under ultrasound guidance, a 23 gauge 15cm disposable needle (Cook Medical, USA) was trans-abdominally inserted into the ewe's uterus and carefully advanced into the peritoneal cavity of the fetus . To avoid fetal injury, the needle was inserted superior-lateral to the fetal bladder to avoid the umbilical arteries and abdominal organs. After confirming optimal needle placement, a mean amount of 11.75±2.81 x10⁶ cells was slowly injected (60 sec) and appropriate distribution in the peritoneal cavity was observed (table 1). In case of multiple fetuses, each fetus was injected using separate needles.

**Histology & Immunohistochemistry of myocardial infarction**

For TUNEL staining pre-treatment was performed with Enzyme 2 (Leica) for 15 min at 37°C. Detection of Digoxin was done with a biotinylated mouse anti-Digoxigenin antibody (1:500; Sigma Chemical Company). Incubation of TUNEL-Staining was performed on Leica BondMax with Intense-R HRP-Detection-Kit.

**Detection and tracking of CM-DiI⁺/MPIO⁺ human mesenchymal stem cells in the fetal sheep myocardium and other organs**

The heart was processed as follows: After cutting away the apical and basal region, the left ventricular, anterior region (area of injection) was divided into three parts (each approximately 5mm x 5mm) which were then either processed freshly for Flow-Cytometry (part 1), snap frozen for PCR (part 2) or in formaldehyde for immunohistochemistry (part 3).

*Flow Cytometry*

A defined cardiac sample of approximately 5mm x 5mm from the area of injection as well as control samples from non-injected sheep heart was processed for Flow Cytometry analysis. In addition, defined samples of the following organs were immediately analyzed after animal sacrifice: bone marrow, brain, kidneys, liver, lung, spleen and thymus. In addition, a lavage of the intra-peritoneal cavity was performed. As previously described , the samples of each organ were mashed through a 70 µm nylon cell strainer (BD Biosciences, Switzerland) and rinsed with PBS. Afterwards, the cell suspension was incubated with red blood cell lysis buffer (Sigma Aldrich, Switzerland) for 5 min at 37°C, washed with PBS and fixed in 1% paraformaldehyde. Single cells were analyzed by Flow Cytometry as described. The cells were gated on the forward (FSC-H) and side scatter (SSC-H) dot plot to select mononuclear cells preferentially .

*PCR Analysis*

RNA was isolated from snap-frozen tissues using RNeasy mini kit (Qiagen AG, Switzerland). The quantity and quality was measured with a nanodrop machine (Witec AG, Switzerland). The first-strand cDNA synthesis was generated with 1 µg of total RNA and random-hexamer primer using ThermoScript RT-PCR system (Invitrogen, Switzerland). PCR was performed as described previously . The human specific β -2-microglobulin sequence (upstream primer: 5'-GTGTCTGGGTTTCATCCATC-3', downstream primer: 5′–GGCAGGCATACTCATCTTTT–3′) and the housekeeping gene β -actin (upstream primer: 5′–CGGGACCTGACTGACTAC–3′, downstream primer: 5′–GAAGGAAGGCTGGAAGAG–3′) were amplified with the following conditions: initialization at 95°C for 9 min followed by 45 cycles of denaturation at 95°C for 30 s, annealing at 55°C for 30 s, elongation at 72°C for 15 s and final elongation at 72°C for 5 min. The PCR product was analyzed on a 2% agarose gel stained with GelRed nucleic acid stain (Chemie Brunschwig, Switzerland). Afterwards, DNA fragments of 163 bp for β -2-microglobulin or 252 bp for β -actin were detected.

*Immunohistochemistry*

To assess the presence and the survival of human mesenchymal stem cells in the fetal myocardium the following immunohistochemical analysis was performed after dewaxing process of the paraffin slides: human cell-specific MHC-1 (1:500; Epitomics, USA), rabbit anti-FITC (1:1000; Invitrogen BV + Research Genetics, Switzerland), human ALU Sequence (ALU Probe; Leica Microsystems AG, Switzerland) and double staining for rabbit anti-FITC + human ALU Sequence. Immunohistochemistry and in situ hybridization was performed on Leica BondMax instruments using Refine AP and Refine HRP-Kits (including all buffer solutions from Leica Microsystems Newcastle, Ltd., United Kingdom) according to the manufacturer’s guidelines.

**References**

1. Pittenger MF, Mackay AM, Beck SC, Jaiswal RK, Douglas R, Mosca JD, Moorman MA, Simonetti DW, Craig S, Marshak DR. Multilineage potential of adult human mesenchymal stem cells. Science 1999;284:143-147.

2. Raschzok N, Billecke N, Kammer NN, Morgul MH, Adonopoulou MK, Sauer IM, Florek S, Becker-Ross H, Huang MD. Quantification of cell labeling with micron-sized iron oxide particles using continuum source atomic absorption spectrometry. Tissue Eng Part C Methods 2009;15:681-686.

3. Raschzok N, Morgul MH, Pinkernelle J, Vondran FW, Billecke N, Kammer NN, Pless G, Adonopoulou MK, Leist C, Stelter L, Teichgraber U, Schwartlander R, Sauer IM. Imaging of primary human hepatocytes performed with micron-sized iron oxide particles and clinical magnetic resonance tomography. J Cell Mol Med 2008;12:1384-1394.

4. Raschzok N, Muecke DA, Adonopoulou MK, Billecke N, Werner W, Kammer NN, Zielinski A, Behringer PA, Ringel F, Huang MD, Neuhaus P, Teichgraber U, Sauer IM. In vitro evaluation of magnetic resonance imaging contrast agents for labeling human liver cells: implications for clinical translation. Mol Imaging Biol 2011;13:613-622.

5. Schoeberlein A, Holzgreve W, Dudler L, Hahn S, Surbek DV. In utero transplantation of autologous and allogeneic fetal liver stem cells in ovine fetuses. Am J Obstet Gynecol 2004;191:1030-1036.

6. Schoeberlein A, Holzgreve W, Dudler L, Hahn S, Surbek DV. Tissue-specific engraftment after in utero transplantation of allogeneic mesenchymal stem cells into sheep fetuses. Am J Obstet Gynecol 2005;192:1044-1052.

7. Shaw SW, Bollini S, Nader KA, Gastadello A, Mehta V, Filppi E, Cananzi M, Gaspar HB, Qasim W, De Coppi P, David AL. Autologous transplantation of amniotic fluid-derived mesenchymal stem cells into sheep fetuses. Cell Transplant 2011;20:1015-1031.

8. Liechty KW, MacKenzie TC, Shaaban AF, Radu A, Moseley AM, Deans R, Marshak DR, Flake AW. Human mesenchymal stem cells engraft and demonstrate site-specific differentiation after in utero transplantation in sheep. Nat Med 2000;6:1282-1286.
